# 4H-SiC Drift Step Recovery Diode with Super Junction for Hard Recovery

**DOI:** 10.3390/ma14030684

**Published:** 2021-02-02

**Authors:** Xiaoxue Yan, Lin Liang, Xinyuan Huang, Heqing Zhong, Zewei Yang

**Affiliations:** State Key Laboratory of Advanced Electromagnetic Engineering and Technology, School of Electrical and Electronic Engineering, Huazhong University of Science and Technology, Wuhan 430074, China; d201980476@hust.edu.cn (X.Y.); m201971424@hust.edu.cn (X.H.); zhongheqing@hust.edu.cn (H.Z.); m202071575@hust.edu.cn (Z.Y.)

**Keywords:** silicon carbide (SiC), drift step recovery diode (DSRD), super junction (SJ), hard recovery, pulsed power switch, diode

## Abstract

Silicon carbide (SiC) drift step recovery diode (DSRD) is a kind of opening-type pulsed power device with wide bandgap material. The super junction (SJ) structure is introduced in the SiC DSRD for the first time in this paper, in order to increase the hardness of the recovery process, and improve the blocking capability at the same time. The device model of the SJ SiC DSRD is established and its breakdown principle is verified. The effects of various structure parameters including the concentration, the thickness, and the width of the SJ layer on the electrical characteristics of the SJ SiC DSRD are discussed. The characteristics of the SJ SiC DSRD and the conventional SiC DSRD are compared. The results show that the breakdown voltage of the SJ SiC DSRD is 28% higher than that of the conventional SiC DSRD, and the dv/dt output by the circuit based on SJ SiC DSRD is 31% higher than that of conventional SiC DSRD. It is verified that the SJ SiC DSRD can achieve higher voltage, higher cut-off current and harder recovery characteristics than the conventional SiC DSRD, so as to output a higher dv/dt voltage on the load.

## 1. Introduction

In the early 1980s [1], the Ioffe Physical Technical Institute proposed a nanosecond switch drift step recovery diode (DSRD). It is a kind of pulse power switch based on the plasma principle, which can operate in the inductive energy storage circuit. The DSRD-based pulse generator is widely used in ground penetrating radar [2], electromagnetic pulse radar [3,4], accelerator [5], igniter [6], ultra-high speed broadband beam deflection [7], etc. In order to improve its operating voltage and current and switching speed, DSRDs based on new materials such as silicon carbide (SiC) and gallium arsenide (GaAs) were prepared. GaAs can produce DSRD with a faster switching speed than SiC because its electron mobility is about 10 times that of SiC. The reference [8] reported a GaAs DSRD with a blocking voltage of 150 V and a switching speed of 100 ps. However, SiC has more advantages than GaAs at higher voltage levels, because its bandgap is more than 2 times that of GaAs, and its critical breakdown electric field is 7–8 times that of GaAs. DSRD based on SiC was proposed in 2002 [9]. In terms of electrical characteristics, the switching speed of SiC DSRD can reach 2–4 times of Si DSRD due to its high critical breakdown electric field and fast saturated electron drift velocity, and the breakdown voltage can reach 10 times of Si DSRD under the same drift layer thickness.

In 2003 [10], I. V. Grekhov et al. reported a SiC DSRD with an area of 2.2 × 10^−3^ cm^2^, which can output a voltage with a rise time of about 4 ns and a pulse amplitude of 400 V. In 2012 [11], P. A. Ivanov et al. fabricated devices with an area of 3.9 × 10^−3^ cm^2^ which can output a voltage with a rise time of 2 ns. In 2015 [12], A.V. Afanasyev et al. reported that a SiC DSRD with a breakdown voltage of 1 kV and an active area of 1 × 10^−2^ cm^2^, which can output a pulse voltage with an amplitude of 1125 V and a rise time of fewer than 2 ns. In 2020 [13], Ruize Sun et al. output a voltage pulse with a leading edge rise time of 1.8 ns (20–90%) on the load. The die area of SiC DSRD needs to be further increased to adapt to higher current applications. However, the increase of area leads to the increase of junction capacitance, which leads to the decrease of switching speed. Therefore, it is very important to obtain a faster switching speed and a larger operating current in the same area without reducing breakdown voltage.

In order to solve the above problem, in addition to choosing materials with better physical properties, such as SiC, the device structure can also be improved. Super junction (SJ) technology is a kind of technology that uses alternating p-type doping region and n-type doping region structure to realize charge compensation and act as voltage withstand layer, so as to obtain a low specific on-resistance and high breakdown voltage at the same time. In 1984 [14], D. J. Coe proposed the method of using alternate pn junction instead of low doped drift layer as voltage withstand layer in laterally diffused metal–oxide–semiconductor field-effect transistor (LDMOSFET) for the first time. In 1991 [15], Xingbi Chen proposed the idea of using multiple pn junction structures as drift layers in longitudinal power devices (especially longitudinal MOSFETs), which is called the Composite Buffer layer. In 1997 [16], T. Fujihira formally proposed the concept of SJ structure, which is a summary of the above ideas. SJ is often used in unipolar devices, such as MOSFET [15,17,18], schottky barrier diode (SBD) [19], and junction field-effect transistor (JFET) [20], to coordinate the contradiction between on-state resistance and breakdown voltage. However, at the same time, it is found that the recovery characteristics of MOSFET bulk diodes become worse after using SJ, that is, a higher current peak value appears during reverse recovery [21]. However, this phenomenon may be beneficial to the dynamic characteristics of DSRD. On one hand, as a kind of current-breaker, a larger reverse current peak on DSRD means that its working current (i.e., cut-off current) can be larger. On the other hand, in the pulse generator based on DSRD, the load is often in parallel with DSRD. The shutdown process is essentially a process of current switching from DSRD to load. Ideally, the greater the reverse current of DSRD in the shutdown process, the greater the current on the load and the greater the load voltage (*V*_peak_). In addition, it is worth noting that the rising speed of the voltage on the load largely depends on the current turn-off speed of DSRD, that is, the faster the reverse current of DSRD drops, the shorter the rising time (*t*_r_) of the load voltage and the greater the leading edge rise rate (dv/dt).

In this paper, the SJ structure is introduced in the SiC DSRD for the first time to increase the hardness of the recovery process, and improve the blocking capability at the same time. The device model of the SJ SiC DSRD is established by TCAD. Based on this model, the rectangular electric field distribution of SJ SiC DSRD during the reverse breakdown is analyzed. The dynamic characteristics of the SJ SiC DSRD and the conventional SiC DSRD under different structure parameters and different external circuit conditions are compared.

## 2. Methods and Results

**Figure 1:**
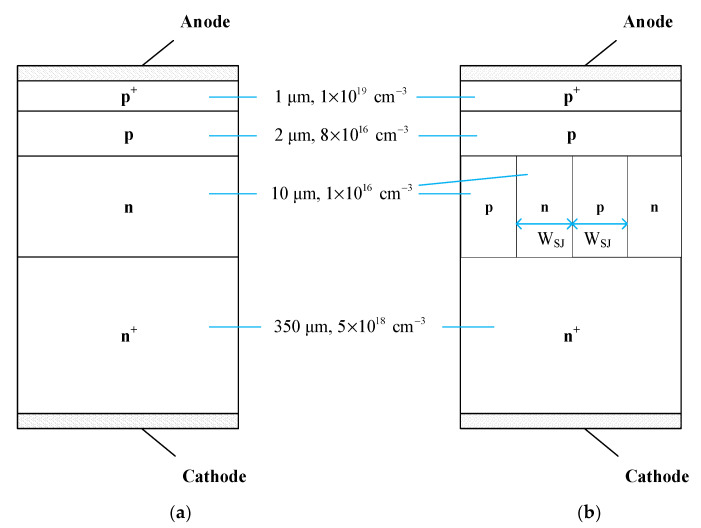
Profiles of (**a**) conventional SiC DSRD and (**b**) SJ SiC DSRD.

DSRD is a kind of current breaker based on the drift step recovery effect. There are two common structures: p^+^-p-n^+^ and p^+^-p-n-n^+^. For high-voltage DSRD, p^+^-p-n-n^+^ has more advantages than p^+^-p-n^+^ because it does not need mesa deep etching and the saturated drift velocity of n-type drift layer is higher [22]. In this paper, the p^+^-p-n-n^+^ four-layer structure is selected as the basic structure of 4H-SiC DSRD, as shown in Figure 1a, in which the moderately doped p layer is used as the charge storage layer, and the lightly doped n layer is used as the voltage withstand layer. SJ is a structure composed of p-type semiconductor and n-type semiconductor alternately to replace the original voltage withstand region. A longitudinal structure of SiC DSRD with SJ structure is shown in Figure 1b. The structure models of conventional SiC DSRD and SJ SiC DSRD are established by simulation, and the parameters of SiC DSRD and SJ SiC DSRD are identical, except for the structure of the drift layer: the concentration of p^+^ emitter layer is 1 × 10^19^ cm^−3^, and the thickness is 1 μm; the concentration of p-base layer is 8 × 10^16^ cm^−3^, and the thickness is 2 μm; the concentration of n^+^ substrate layer is 5 × 10^18^ cm^−3^, and the thickness is 350 μm; the concentration (*N*_nb_) of drift region (referring to n base layer of conventional SiC DSRD or p-pillar and n-pillar of SJ SiC DSRD) is 1 × 10^16^ cm^−3^, and the thickness (*T*_nb_) is 10 μm. The widths (*W*_SJ_) of p-pillar and n-pillar of DSRD are equal, which are 2 μm. Each layer is uniformly doped. The front side is the anode and the back side is the cathode. It is also necessary to design a termination structure in the actual preparation, and silicon dioxide or silicon nitride can be used as the terminal passivation layer [23].

In order to analyze the breakdown mechanism of SJ SiC DSRD, the electric field distribution of conventional SiC DSRD and SJ SiC DSRD during reverse breakdown was explored. According to the reference [24], bandgap narrowing in heavily doped emitters and incomplete ionization of impurities have a great influence on the switching characteristics of SiC DSRD. Therefore, the above physical effects are considered in the simulation. In addition, high field saturation, Auger recombination and Shockley-Read-Hall recombination [25] are considered in the model, too. The electrical parameters and characteristics of conventional SiC DSRD and SJ SiC DSRD are analyzed by numerically solving Poisson equation, continuity equation and transport equation.

**Figure 2:**
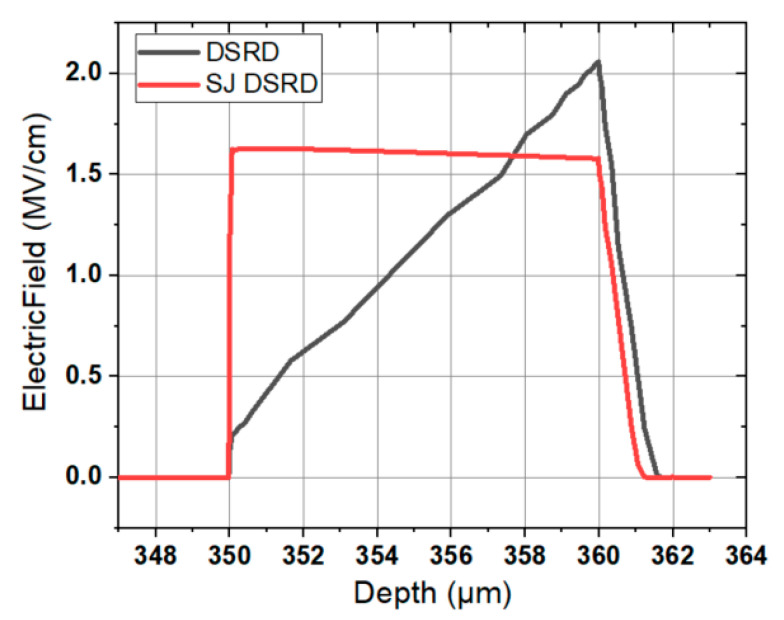
Electric field distributions in drift layer of conventional SiC DSRD and SJ SiC DSRD. (*T*_nb_ of 10 μm, *N*_nb_ of 1 × 10^16^ cm^−3^).

As shown in Figure 2, the electric field distribution of the two devices under reverse breakdown is compared, where the SJ SiC DSRD is the electric field distribution along the interface of p-pillar and n-pillar. When the external reverse voltage is applied, the p-pillar and n-pillar regions in the voltage withstand layer are depleted, and the negative and positive charges are generated by the ionization of the acceptor and donor. The positive charges in the n-pillar generate the electric field terminating in the p-pillar along the transverse direction. With the increase of reverse voltage, the transverse pn junction and longitudinal pn junction interact, and the electric field broadens in the transverse and longitudinal directions, so that the total electric field can change from the traditional triangular distribution (non through) or trapezoidal distribution (through) to rectangular distribution, thus increasing the breakdown voltage of the device. The reverse breakdown voltage of the SJ SiC DSRD in Figure 2 is 1606 V, which is 28% higher than that of the conventional SiC DSRD (1257 V).

**Figure 3:**
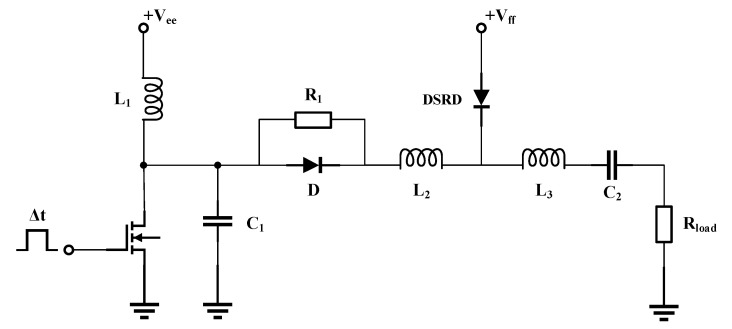
Pulse test circuit based on SiC DSRD.

Theoretically, the area of the n-pillar in the voltage withstand layer of SiC DSRD becomes half of the original area after the introduction of SJ. Under the premise of equal doping concentration, the carrier concentration is reduced by half, so it has a larger peak current and a faster current drop rate in the reverse recovery process. In order to verify the above inference, a pulse test circuit based on DSRD is specially established in simulation, as shown in Figure 3. The detailed operating principle of the circuit can be referred to [26].

**Figure 4:**
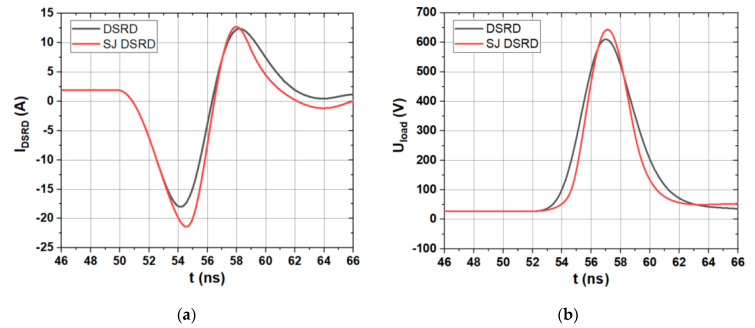
Comparison of (**a**) current waveforms and (**b**) load output voltage waveforms of conventional SiC DSRD and SJ SiC DSRD. (*T*_nb_ of 10 μm, *N*_nb_ of 1 × 10^16^ cm^−3^)

In the simulation, the active area of both devices is 4 × 10^−2^ cm^2^. The circuit parameters are set as *V*_ee_ = 40 V, *V*_ff_ = 30 V, *L*_1_ = 75 nH, *L*_2_ = 65 nH, *L*_3_ = 45 nH, *C*_1_ = 100 pF, *C*_2_ = 1 μF, Δt = 50 ns, *R*_1_ = 20 Ω, *R*_load_ = 50 Ω. As shown in Figure 4a, the current waveforms of the conventional SiC DSRD and the SJ SiC DSRD are compared. It can be seen that the reverse peak current of SJ SiC DSRD is larger than that of conventional SiC DSRD, and the reverse current of SJ SiC DSRD decreases faster than that of conventional SiC DSRD. Figure 4b shows the comparison of load voltage waveforms between conventional SiC DSRD and SJ SiC DSRD. It can be seen that the SJ SiC DSRD can output pulse voltage with a faster-rising speed and higher peak value than the conventional SiC DSRD. The parameters are summarized as shown in Table 1, where BV is the breakdown voltage, *V*_peak_ is the peak voltage, *t*_r_ is the rise time of load voltage leading edge, which usually refers to the time from 10% of *V*_peak_ to 90% of *V*_peak_, dv/dt is average voltage change rate in *t*_r_ as shown in Equation (1).

(1) $$dv/dt=(0.9-0.1)V_{peak}/t_{r}$$

It can be seen that the *t_r_* of SJ SiC DSRD is 18.5% less than that of conventional SiC DSRD, and the dv/dt is 29% higher than that of conventional SiC DSRD.

## 3. Discussion

### 3.1. Influence of Structure Parameters on Blocking Characteristics

**Figure 5:**
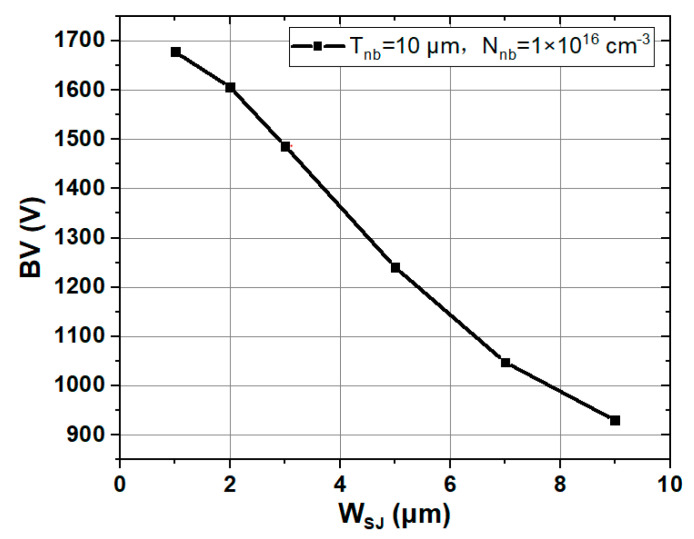
BV of SJ SiC DSRD with different *W*_SJ_. (*T*_nb_ of 10 μm, *N*_nb_ of 1 × 10^16^ cm^−3^).

By introducing an electric field in the transverse direction, the SJ structure affects the distribution of the longitudinal electric field, and finally changes the breakdown voltage. The voltage is essentially affected by *W*_SJ_, *T*_nb_ and *N*_nb_. As shown in Figure 5, it is the BV curve formed by connecting the discrete simulation values obtained under different *W*_SJ_. With the increase of the *W*_SJ_, the BV decreases rapidly. The reason is that with the increase of *W*_SJ_, the adjustment ability of the transverse electric field to the total electric field is weakened, and the total electric field distribution is no longer rectangular.

**Figure 6:**
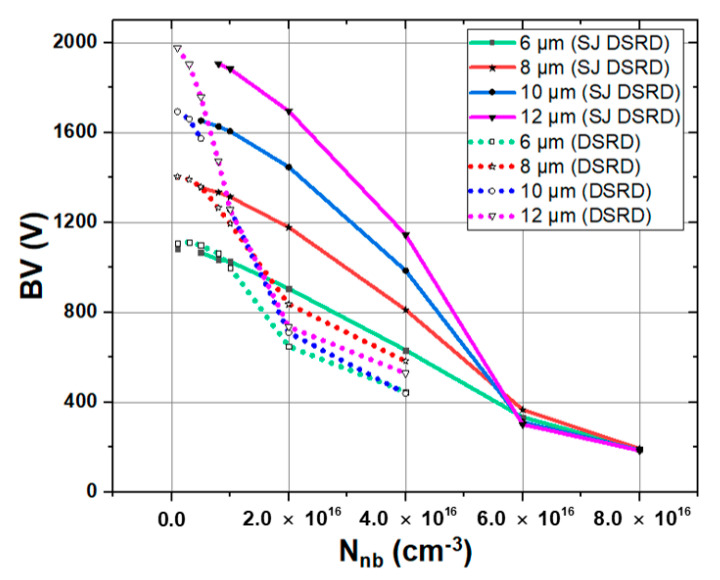
BV of conventional SiC DSRD and SJ SiC DSRD under different *N*_nb_ and *T*_nb_.

**Figure 7:**
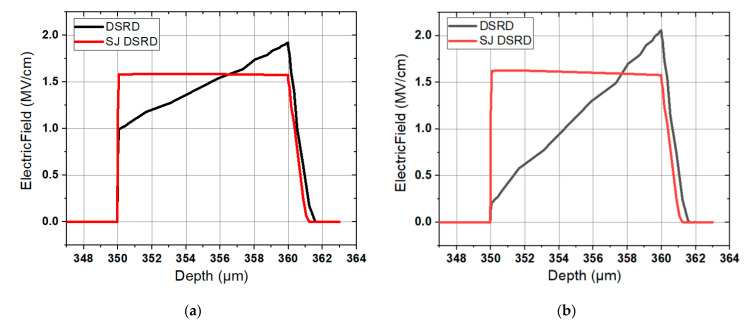
Comparison of electric field distributions of conventional SiC DSRD and SJ SiC DSRD with drift layer concentrations of (**a**) 5 × 10^15^ cm^−3^ and (**b**) 1 × 10^16^ cm^−3^ respectively.

As shown in Figure 6, the breakdown voltages of conventional SiC DSRD and SJ SiC DSRD are compared under different drift layer concentrations and thicknesses. Note that in order to facilitate observation and analysis, the breakdown voltages are connected into curves. As can be seen from the figure that the BV of both devices decreases with the increase of *N*_nb_, but the BV of SJ SiC DSRD always keeps the trend of being larger than or equal to that of conventional SiC DSRD. Taking the concentrations of 5 × 10^15^ cm^−3^ and 1 × 10^16^ cm^−3^ as examples, as shown in Figure 7a,b, the electric field distribution of conventional SiC DSRD and SJ SiC DSRD with drift layer concentrations of 5 × 10^15^ cm^−3^ and 1 × 10^16^ cm^−3^ are compared. The area formed by the electric field curve and the horizontal axis represents the breakdown voltage. It can be seen that for the SJ SiC DSRD, the breakdown voltages at the concentration of 5 × 10^15^ cm^−3^ and 1 × 10^16^ cm^−3^ are almost the same; for the conventional SiC DSRD, when the concentration changes from 5 × 10^15^ cm^−3^ to 1 × 10^16^ cm^−3^, the electric field distribution tends from the trapezoidal distribution with larger punch-through to the trapezoidal distribution with smaller punch-through, that is, the breakdown voltage decreases rapidly. In conclusion, when the concentration is 1 × 10^16^ cm^−3^, the increase of breakdown voltage is better than that when the concentration is 5 × 10^15^ cm^−3^. In addition, it can be seen from Figure 6 that the breakdown voltage of SJ SiC DSRD gradually increases with the increase of the thickness of the drift layer at the same doping concentration; at the same time, in the appropriate doping concentration range, the greater the thickness of the drift layer, the higher the degree of improvement of the breakdown voltage of the SJ SiC DSRD.

### 3.2. Influence of Structure Parameters on Dynamic Characteristics

For SiC DSRD, one of the most important applications is to output a fast-rising high voltage pulse on load for the pulse generator. In this process, the most important parameters are the rise time *t*_r_ (usually between 10% and 90% of the pulse voltage amplitude) and the peak value *V*_peak_. In some applications, small *t*_r_ and large *V*_peak_ are required to achieve high dv/dt. The quality of the load pulse is directly affected by the switching characteristics of DSRD. According to the operating principle of DSRD, the faster the reverse current of DSRD decreases, the shorter the rise time of the output pulse voltage on the load; the larger the reverse current peak value of DSRD, the larger the output voltage peak value on the load. Based on the pulse generator circuit shown in Figure 3, we focus on the influence of different structure parameters on the output pulse of conventional SiC DSRD and SJ SiC DSRD.

**Figure 8:**
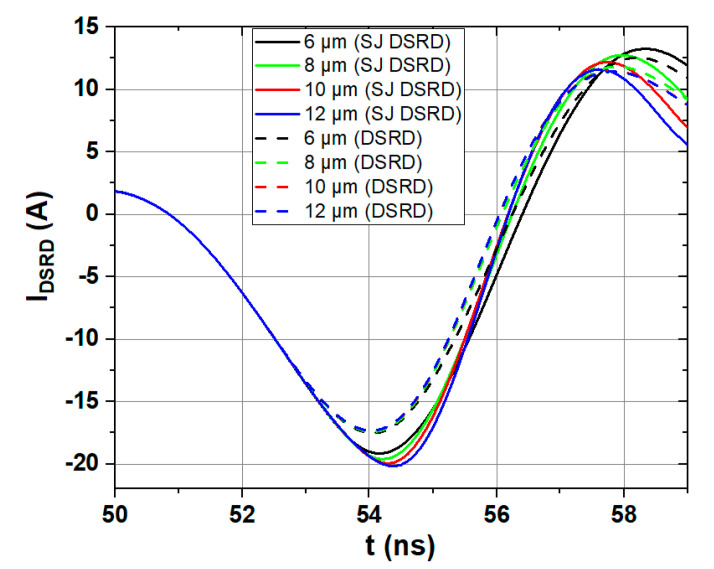
Current waveforms of conventional SiC DSRD and SJ SiC DSRD with *T*_nb_ of 6 μm, 8 μm, 10 μm, and 12 μm. (*T*_nb_ of 7 × 10^15^ cm^−3^, the same circuit conditions)

The current waveforms of conventional SiC DSRD and SJ SiC DSRD with *T*_nb_ of 6 μm, 8 μm, 10 μm and 12 μm are shown in Figure 8. It can be seen that the peak value of the reverse current of the SJ SiC DSRD is larger than that of the conventional SiC DSRD, and the decrease speed of the reverse current is faster than that of conventional SiC DSRD. The *V*_peak_, *t*_r_ and dv/dt of the output voltage pulse on the load in the pulse circuit of the two devices are shown in Table 2, where *T*_nb_ is the thickness of the drift layer. It can be seen that the *V*_peak_ of SJ SiC DSRD is generally larger and *t*_r_ is smaller than that of conventional SiC DSRD. For dv/dt, the improved range of SJ SiC DSRD can reach 31% compared with conventional SiC DSRD.

**Figure 9:**
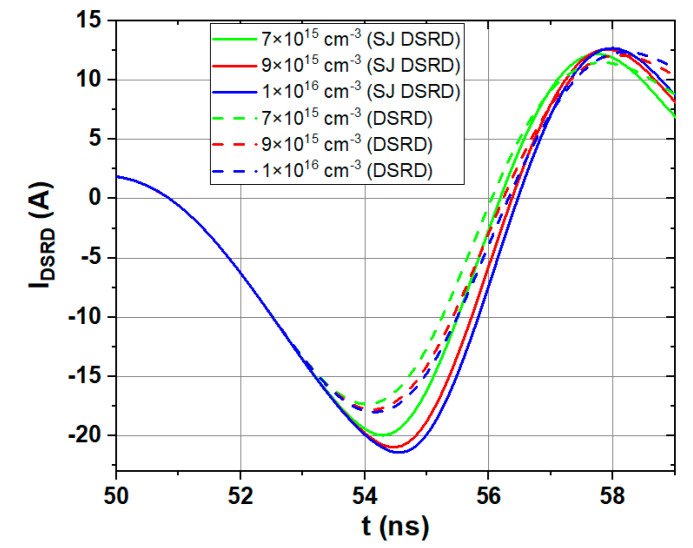
Current waveforms of conventional SiC DSRD and SJ SiC DSRD with *N*_nb_ of 7 × 10^15^ cm^−3^, 9 × 10^15^ cm^−3^, and 1 × 10^16^ cm^−3^. (*T*_nb_ of 10 μm, the same circuit conditions)

Using the same method, the influence of *N*_nb_ on the dynamic characteristics of the conventional SiC DSRD and the SJ SiC DSRD are discussed. The current waveforms of them with *N*_nb_ of 7 × 10^15^ cm^−3^, 9 × 10^15^ cm^−3^ and 1 × 10^16^ cm^−3^ are shown in Figure 9. It can be seen that the SJ SiC DSRD has larger reverse peak currents and harder recovery characteristics. The output voltage parameters of the two devices under these three parameters are summarized in Table 3. It can be seen that the SJ SiC DSRD can output voltage pulses with a higher peak, shorter rise time, and larger dv/dt than the conventional SiC DSRD. When the *N*_nb_ is 1 × 10^16^ cm^−3^, the increase of dv/dt can be as high as 29%. Combined with the above breakdown analysis, when *N*_nb_ is 1 × 10^16^ cm^−3^, the breakdown voltage of SJ SiC DSRD is 1606 V, which is 28% higher than that of conventional SiC DSRD 1257 V.

### 3.3. Influence of External Circuit Parameters on Dynamic Characteristics

In addition, it is well known that the *V*_peak_ and dv/dt of the pulse voltage that DSRD can output on the load are also related to the forward pumping time of the external circuit. Therefore, the dynamic characteristics of conventional SiC DSRD and SJ SiC DSRD under different forward pumping time are also simulated. In the simulation, the parameters of the devices are the same except that one uses a separate n-type region and the other uses a SJ composed of p-type and n-type alternately as their drift layer. As shown in Table 4, when Δt (forward pumping time) is 30 ns, 40 ns, 50 ns, 60 ns, 80 ns, 100 ns, 120ns, 140 ns, 160 ns, 180 ns and 200 ns, the output voltage parameters of conventional SiC DSRD and SJ SiC DSRD are compared. It can be seen that the *V*_peak_ and dv/dt of SJ SiC DSRD are larger, and the *t*_r_ is smaller than those of conventional SiC DSRD at any Δt. Specifically, the dv/dt can be increased by 12–23%.

## 4. Conclusions

The SJ structure is introduced in the SiC DSRD for the first time in this paper. Static simulations show that the breakdown voltage of SJ SiC DSRD is affected by the doping concentration, thickness and width of the SJ. The breakdown voltage increases with the decrease of SJ doping concentration, the increase of SJ thickness, and the decrease of SJ width. What’s more, SJ SiC DSRD can withstand higher reverse breakdown voltage than conventional SiC DSRD under the same conditions. When the drift region thickness is 10 μm and the concentration is 1 × 10^16^ cm^−3^, the breakdown voltage of SJ SiC DSRD is 28% higher than that of conventional SiC DSRD.

Dynamic simulations show that SJ SiC DSRD can cut off a larger reverse current and has a shorter reverse current fall time than conventional SiC DSRD. In other words, SJ can provide a higher operating current and improve the recovery hardness of SiC DSRD at the same time. Because of the above advantages, the pulse circuit based on SJ SiC DSRD can output voltage pulses with larger amplitude and steeper rising front compared with the pulse circuit based on conventional SiC DSRD. When the doping concentration of the drift region is 7 × 10^15^ cm^−3^ and the thickness is 12 μm, the dv/dt of SJ SiC DSRD is 31% higher than that of conventional SiC DSRD.

## Figures and Tables

**Table 1:** Comparison of parameter characteristics between conventional SiC DSRD and SJ SiC DSRD.

Device	BV (V)	V_peak_ (V)	t_r_ (ns)	dv/dt (V/ns)
Conventional SiC DSRD	1257	610	2.7	181
SJ SiC DSRD	1606 (28% higher)	643	2.2 (18.5% faster)	234 (29% higher)

**Table 2:** Comparison of output pulse parameters of conventional SiC DSRD and SJ SiC DSRD with *T*_nb_ of 6 μm, 8 μm, 10 μm, and 12 μm.

T_nb_ (μm)	V_peak_ (V)		t_r_ (ns)		dv/dt (V/ns)	
	Conventional SiC DSRD	SJ SiC DSRD	Conventional SiC DSRD	SJ SiC DSRD	Conventional SiC DSRD	SJ SiC DSRD
6	604	603	2.7	2.5	179	193 (8% higher)
8	629	635	2.5	2.3	201	221 (10% higher)
10	636	654	2.5	2.1	204	249 (22% higher)
12	635	662	2.5	2.0	203	265 (31% higher)

**Table 3:** Comparison of output pulse parameters of conventional SiC DSRD and SJ SiC DSRD with *N*_nb_ of 7 × 10^15^ cm^−3^, 9 × 10^15^ cm^−3^, and 1 × 10^16^ cm^−3^. (*T*_nb_ of 10 μm, the same circuit conditions).

N_nb_ (cm^−3^)	V_peak_ (V)		t_r_ (ns)		dv/dt (V/ns)	
	Conventional SiC DSRD	SJ SiC DSRD	Conventional SiC DSRD	SJ SiC DSRD	Conventional SiC DSRD	SJ SiC DSRD
7 × 10^15^	636	654	2.6	2.2	196	238 (21% higher)
9 × 10^15^	619	648	2.6	2.3	190	225 (18% higher)
1 × 10^16^	609	643	2.7	2.2	181	234 (29% higher)

**Table 4:** Output voltage comparison of conventional SiC DSRD and SJ SiC DSRD when Δt is 30 ns, 40 ns, 50 ns, 60 ns, 80 ns, 100 ns, 120 ns, 140 ns, 160 ns, 180 ns, and 200 ns respectively. (*T*_nb_ of 10 μm, *N*_nb_ of 7 × 10^15^ cm^−3^).

Δt (ns)	V_peak_ (V)		t_r_ (ns)		dv/dt (V/ns)	
	Conventional SiC DSRD	SJ SiC DSRD	Conventional SiC DSRD	SJ SiC DSRD	Conventional SiC DSRD	SJ SiC DSRD
30	367	383	3.1	2.8	95	109 (15% higher)
40	502	522	2.6	2.3	154	182 (18% higher)
50	636	654	2.5	2.1	204	249 (22% higher)
60	766	782	2.4	2.1	255	298 (17% higher)
80	1015	1029	2.3	2.0	353	412 (17% higher)
100	1247	1266	2.2	2.0	453	506 (12% higher)
120	1350	1488	2.2	2.1	491	567 (15% higher)
140	1442	1562	2.2	2.0	524	625 (19% higher)
160	1473	1628	2.2	2.0	535	651 (22% higher)
180	1507	1628	2.2	2.0	548	651 (19% higher)
200	1534	1723	2.3	2.1	534	656 (23% higher)

## Data Availability

Detailed data are available on request from the corresponding author.
